# Health, social, behavioral, and labor correlates of quality of life in a well population: a contribution to construct validity of the WHOQOL-BREF

**DOI:** 10.1590/0102-311XEN043325

**Published:** 2025-12-01

**Authors:** Luiz Antônio Bastos Camacho, Victor Chagas Matos, Leidjaira Lopes Juvanhol, Maria de Jesus Mendes da Fonseca, Eduardo Faerstein

**Affiliations:** 1 Escola Nacional de Saúde Pública Sergio Arouca, Fundação Oswaldo Cruz, Rio de Janeiro, Brasil.; 2 Departamento de Nutrição e Saúde, Universidade Federal de Viçosa, Viçosa, Brasil.; 3 Instituto Oswaldo Cruz, Fundação Oswaldo Cruz, Rio de Janeiro, Brasil.; 4 Instituto de Medicina Social, Universidade do Estado do Rio de Janeiro, Rio de Janeiro, Brasil.

**Keywords:** Quality of Life, Occupational Health, Psychological Distress, Qualidade de Vida, Saúde Ocupacional, Angústia Psicológica, Calidad de Vida, Salud Laboral, Distrés Psicológico

## Abstract

The study aims to assess the hypothetical construct underlying the WHOQOL-BREF scale of quality of life in the working environment, wherein individuals are reasonably healthy. We analyzed cross-sectional data from civil servants at university campuses in Rio de Janeiro, Brazil, which focused on social determinants of health possibly related to quality of life. We simultaneously assessed the association of quality of life domains with physical and mental health, physical activity, eating habits, stress and social support at work, situations of discrimination, and socioeconomic status using structural equation modeling. Most of the 3,574 participants were non-manual workers (57%), woman (56%), with median age of 42 years, and 42% had a university degree. The association of psychological distress stood out from other covariates in all four domains of quality of life, particularly the psychological domain. The direct association between socioeconomic status and the environmental domain, the inverse association of recent morbidity to the physical domain, and the inverse association of discrimination with all domains were also noteworthy. The results strengthened the construct validity from factor analysis of WHOQOL-BREF, with additional evidence from the association with constructs related to quality of life obtained in a study of social determinants of health. There seems to be space to expand the use of WHOQOL-BREF to provide valid inferences on the quality of life of workers, targeting individuals whose very low scores might signal the need for support, and to assess changes in quality of life following interventions in groups of workers.

## Abstract (pt)

O objetivo do estudo foi avaliar o construto hipotético subjacente à escala WHOQOL-BREF de qualidade de vida no ambiente de trabalho em que os indivíduos são razoavelmente saudáveis. Analisamos dados transversais provenientes de servidores públicos que trabalham em *campi* universitários no Rio de Janeiro, Brasil, focando em determinantes sociais da saúde possivelmente relacionados à qualidade de vida. Avaliamos simultaneamente a associação dos domínios da qualidade de vida com saúde física e mental, atividade física, hábitos alimentares, estresse e apoio social no trabalho, situações de discriminação e condição socioeconômica, utilizando modelagem de equações estruturais. A maioria dos 3.574 participantes eram trabalhadores não manuais (57%) e do sexo feminino (56%). A idade mediana foi de 42 anos e 42% tinham diploma universitário. A associação de sofrimento psicológico se destacou das outras covariáveis ​​em todos os quatro domínios da qualidade de vida, particularmente no domínio psicológico. Também identificou-se associação direta entre condição socioeconômica e o domínio ambiental, associação inversa de morbidade recente com o domínio físico e associações inversas de discriminação com todos os domínios. Os resultados fortaleceram a validade de construto da análise fatorial do WHOQOL-BREF, com evidências adicionais da associação aos construtos relacionados à qualidade de vida obtidas em estudo sobre os determinantes sociais da saúde. Parece haver espaço para expandir o uso do WHOQOL-BREF para fornecer inferências válidas sobre a qualidade de vida de trabalhadores, focando em indivíduos cujas pontuações muito baixas podem sinalizar a necessidade de apoio, e para avaliar mudanças na qualidade de vida após intervenções em grupos de trabalhadores.

## Abstract (es)

El objetivo del estudio fue evaluar el constructo hipotético subyacente a la escala WHOQOL-BREF de calidad de vida en el entorno laboral, en el que las personas son razonablemente sanas. Analizamos datos transversales de funcionarios públicos de *campi* universitarios de Rio de Janeiro (Brasil), centrándonos en los determinantes sociales de la salud que podrían estar relacionados con la calidad de vida. Evaluamos simultáneamente la asociación de los ámbitos de la calidad de vida con la salud física y mental, la actividad física, los hábitos alimenticios, el estrés y el apoyo social en el trabajo, las situaciones de discriminación y el estatus socioeconómico utilizando modelos de ecuaciones estructurales. La mayoría de los 3.574 participantes eran trabajadores no manuales (57%), mujeres (56%), con una edad media de 42 años, y el 42% tenía un título universitario. La asociación del sufrimiento psicológico destacó sobre otras covariables en los cuatro ámbitos de la calidad de vida, especialmente en el ámbito psicológico. También cabe destacar la asociación directa entre el estatus socioeconómico y el ámbito medioambiental, la asociación inversa entre la morbilidad reciente y el ámbito físico, y la asociación inversa entre la discriminación y todos los ámbitos. Los resultados reforzaron la validez del constructo a partir del análisis factorial del WHOQOL-BREF, con pruebas adicionales de la asociación con constructos relacionados con la calidad de vida obtenidos en un estudio sobre los determinantes sociales de la salud. Parece haber margen para ampliar el uso del WHOQOL-BREF con el fin de obtener inferencias válidas sobre la calidad de vida de los trabajadores, centrándose en aquellas personas cuyas puntuaciones muy bajas podrían indicar la necesidad de apoyo, y para evaluar los cambios en la calidad de vida tras las intervenciones en grupos de trabajadores.

## Introduction

The World Health Organization (WHO) has defined quality of life as “*individuals’ perceptions of their positions in life in the context of the culture and value systems in which they live, and in relation to their goals, expectations, standards, and concerns*” ^1^ (p. 1405). Under its auspices, the WHOQOL Group developed a 100-question instrument providing a multi-dimensional profile of scores across 24 sub-domains (facets) within the following domains of quality of life: physical ability, psychological domain, degree of independence, social relationships, environment, and spirituality/religion/beliefs ^2^.

Intended uses for the WHOQOL instrument included the assessment of changes in the quality of life of an individual following health interventions and in epidemiological research ^2^. An abbreviated version - WHOQOL-BREF - was developed by selecting at least one question from each of the 24 facets from four of the original instrument’s domains - physical health, psychological health, social relationships, and environment - and two general questions about quality of life and general health assessment. The WHOQOL-BREF showed a factor structure close to the WHOQOL-100 scale ^3^. It is a self-administered or application-assisted instrument; being multidimensional, each domain should be scored and interpreted separately ^4^ ^,^ ^5^.

In Brazil, a translation and cross-cultural adaptation of the WHOQOL-BREF in outpatients, hospitalized patients, and healthy controls showed good internal consistency and discriminant validity, except in the social relationships’ domain ^6^. The Brazilian version also showed satisfactory internal consistency among civil servants at the State of Rio de Janeiro university campuses ^7^. The construct validity of the WHOQOL has been assessed with factor analysis, which has provided valuable insights and indicated limitations in the performance of the scale. Validation of hypothetical constructs is an ongoing process that can build on studies of association of quality of life scores with variables whose conceptual relation might strengthen the meaning of quality of life.

WHOQOL-BREF domains have demonstrated responsiveness to clinical and social change that is relevant and meaningful, and showed good performance in the discriminant validity of healthy and sick individuals with wide variety of physical, metabolic, and mental disorders ^8^. Most studies on quality of life have targeted patients with chronic conditions, older adults, workers with specific health conditions, migrant, and healthcare workers etc., in which quality of life was associated with peculiar sociodemographic factors ^9^, and occupational hazards ^10^ ^,^ ^11^. The work environment is scarcely mentioned in the many uses of WHOQOL ^2^. Although studies with specific groups are informative, restriction in the variability of attributes in those groups may attenuate measures of association ^12^.

Our analyses include the relationship between the WHOQOL-BREF quality of life domains with health aspects and also with lifestyle and work environment, considering (1) the role attributed to quality of life measures, strongly related but not limited to health-disease aspects, and (2) the challenges in measuring this attribute and in interpreting the measures generated in a group of workers. Beyond content and construct validity of WHOQOL-BREF, a hypothesis test approach could enable higher quality of life to be inferred from higher scores among healthy (or with minor morbidity) workers. By integrating different sources of evidence based on theory, logic, and empirical evidence ^13^ we aimed to contribute to the validation of WHOQOL-BREF in a well population and from a perspective that so far seems to have gotten less attention.

## Methods

### Study design

**Figure 1:**
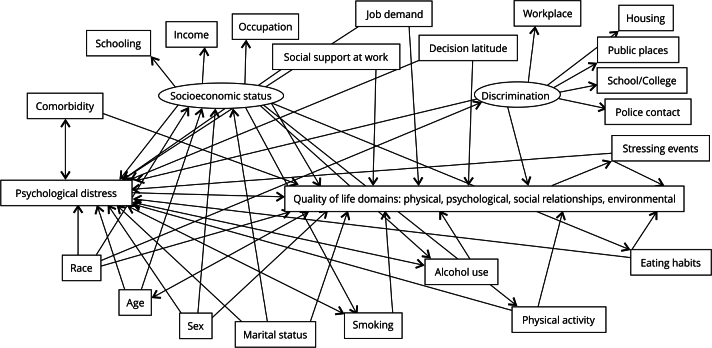
Directed acyclic graph representing the relationships between the domains of quality of life, according to the WHOQOL-BREF, and the attributes of employees of a public university in the State of Rio de Janeiro, Brazil.

This is a cross-sectional study of the relationships between the scores of the domains of quality of life with other elements of physical and mental health, socioeconomic status, life style, stress and social support at work, and situations of discrimination, based on a logical model that gives meaning to the construct quality of life and its implications on the individual’s health (Figure 1).

The objectives of the Pró-Saúde Study, the definition of the study population, and the procedures for the preparation and testing of data collection instruments, and for the selection and approach of participants are detailed elsewhere ^14^. Briefly, the Pró-Saúde Study is a prospective cohort study of non-faculty civil servants at university campuses in Rio de Janeiro, focused primarily on the investigation of social determinants of health. The current analyses used data from the second wave of data collection (2001-2002), conducted with 3,574 civil servants (83% of those eligible) ^15^. The questionnaire was self-completed by participants in the presence of a supervisor and comprised 83 questions, which included the WHOQOL-BREF validated Brazilian Portuguese version ^6^ ^,^ ^7^. The questionnaire also included items regarding physical health (current and previous health problems, hospitalizations, and use of medications), self-rated health status, minor psychiatric morbidity, diet and physical activity, smoking and alcohol consumption, social mobility, stress and accidents at work, stress-producing life events, situations of discrimination, and sociodemographic variables (schooling, job/profession, income, age, and gender).

To guide the analysis of the variables, causal diagrams (directed acyclic graphs - DAG) were constructed with the DAGitty software (http://www.dagitty.net) to represent the hypotheses about the relationships between exposures, outcome, and covariates.

### Study variables and statistical analysis

Four domains of quality of life were measured by WHOQOL-BREF, namely physical, psychological, social relationships, and environmental domains, based on 24 items ^3^. All domains are graded on a Likert scale with scores from 1 to 5, from unfavorable to most favorable, except for three questions that required reverse scoring to reconcile with the other questions in the domain. Scoring followed procedures detailed in WHOQOL user manual ^2^.

The questionnaire included an instrument for screening psychological distress: the *General Health Questionnaire* (GHQ-12) ^16^ ^,^ ^17^, in its validated Brazilian version ^18^. The covariates noncommunicable chronic diseases, recent morbidity, job strain, participant’s experience of stressful events in the previous 12 months, eating habits, discrimination, and socioeconomic status were derived from specific questionnaire items (details are in the Supplementary Material; https://cadernos.ensp.fiocruz.br/static//arquivo/suppl-e00043325_2371.pdf). The analyses also included the practice of physical activity in the previous two weeks, and any work-related accidents in the previous 12 months.

The sample characterization was shown with means and 95% confidence intervals of the scores of the overall quality of life and satisfaction with one’s own health (extra-domain variables) and of each domain, estimated in the categories of the covariates of interest. Statistical analyses were conducted with IBM SPSS Statistics 24 (https://www.ibm.com/).

We used structural equation modeling to evaluate the association of the four domains of quality of life with latent and observed variables. The two extra-domain items (degree of satisfaction with one’s own health and the respondent’s perceived overall quality of life) were not included in structural equation modeling. For the selection of exposures that remained in the final model we adopted a significance level of 10% for the association between exposure and quality of life domains. Different equations for each quality of life domain were simultaneously estimated in the final model. Confounding variables, namely gender, age, physical activity, and eating habits, were the same for the four equations, regardless of the statistical significance of their association with the outcome. Exposure variables, including the latent ones, were allowed to covary within each domain, as were outcomes among themselves. The estimation was made by weighted least squares with mean- and variance-adjusted chi-square test statistic (WSLMV) due to categorical variables and the outcome not being normally distributed. Model fit was evaluated by the root of the mean squared error of approximation (RMSEA), the comparative fit index (CFI), the Tucker-Lewis index (TLI), and the standardized root mean squared residual (SRMR). Given our large sample and numerous variables, we deemed the fit appropriate if CFI and TLI ≥ 0.90, RMSEA ≤ 0.06, and SRMR ≤ 0.08 ^19^. The analyses were conducted with Mplus version 8.8 (https://www.statmodel.com/) ^20^.

### Ethical considerations

This study was an analysis of data collected after approval by the Research Ethics Committee of the State University of Rio de Janeiro (approval n. 224/99). All participants provided written informed consent. Data analysis was conducted without individual identification.

## Results

There was a slight predominance of women (Table 1) with an age distribution similar to the men, with median age of 42 years (interquartile range: 12). Most of them were non-manual routine workers (57%) and had a mean monthly per capita income of less than two minimum wages. A total of 42% of the participants had a university degree (Table 1). Quality of life scores were higher, on average, in the physical domain and substantially lower in the environmental domain. Men had higher mean scores in all quality of life domains but did not differ from women in the overall quality of life score (extra-domain) and in satisfaction with their own health. Younger respondents had slightly higher mean levels of satisfaction with health and quality of life in the physical domain and slightly lower in the social relationships domain. The differences between age groups were negligible in the psychological and environmental domains. The mean scores of quality of life in all its domains, except social relationships, showed a slight tendency to increase with schooling level. Specialized professionals had slightly higher mean scores of quality of life than the other job categories, except in the social relationships domain. The upper categories of average per capita income had higher mean scores in all domains of quality of life and satisfaction with health.

**Table 1:** Means and 95% confidence intervals (95%CI) of quality of life scores (domains and extra-domain items), according to sociodemographic characteristics. Pró-Saúde Study, 2001-2002.

Characteristics	n *	Extra-domain ** [mean (95%CI)]			Domains *** [mean (95%CI)]		
		Overall quality of life	Satisfaction with health	Physical	Psychological	Social relationship	Environment
Total	3,574	17.6 (17.4-17.7)	16.0 (15.8-16.2)	73.7 (73.2-74.2)	70.0 (69.5-70.5)	70.5 (69.9-71.1)	56.6 (56.1-57.0)
Gender							
Woman	1,999	17.5 (17.3-17.7)	15.6 (15.3-15.8)	71.2 (70.5-71.9)	67.9 (67.3-68.6)	68.9 (68.0-69.7)	56.1 (55.5-56.8)
Man	1,575	17.6 (17.4-17.9)	16.6 (16.3-16.9)	76.8 (76.1-77.6)	72.7 (72.0-73.4)	72.6 (71.7-73.5)	57.3 (56.5-58.0)
Age (years)							
24-35	808	17.7 (17.4-18.0)	16.3 (16.0-16.7)	75.3 (74.4-76.3)	70.0 (69.0-71.0)	70.2 (69.0-71.4)	56.8 (55.8-57.8)
36-50	2,185	17.5 (17.3-17.7)	16.0 (15.7-16.2)	73.3 (72.7-74.0)	69.8 (69.2-70.4)	70.1 (69.3-70.9)	56.4 (55.8-57.0)
51+	579	17.7 (17.3-18.1)	15.6 (15.0-16.2)	72.6 (71.2-74.0)	70.8 (69.6-72.1)	72.5 (70.9-74.1)	57.2 (55.9-58.4)
Schooling							
Elementary	771	16.9 (16.6-17.2)	15.4 (14.9-15.9)	71.5 (70.4-72.7)	69.6 (68.5-70.6)	71.7 (70.3-73.2)	53.4 (52.4-54.4)
High school	1,285	17.4 (17.1-17.6)	15.8 (15.5-16.2)	73.4 (72.5-74.2)	69.8 (69.1-70.6)	70.4 (69.4-71.5)	54.2 (53.4-55.0)
University/Postgraduate	1,514	18.0 (17.8-18.3)	16.4 (16.1-16.7)	74.9 (74.2-75.7)	70.4 (69.7-71.1)	69.9 (69.0-70.9)	60.0 (59.3-60.8)
Job category							
Professionals ^#^	1,225	18.1 (17.9-18.4)	16.7 (16.4-17.0)	76.2 (75.4-77.0)	72.1 (71.4-72.8)	71.6 (70.6-72.6)	61.1 (60.3-61.9)
Routine, non-manual	2,012	17.4 (17.2-17.5)	15.5 (15.2-15.8)	72.1 (71.4-72.8)	68.7 (68.1-69.4)	69.6 (68.8-70.4)	54.2 (53.6-54.8)
Supervisors; manual workers	311	16.7 (16.1-17.3)	16.2 (15.4-16.9)	73.5 (71.5-75.4)	69.8 (68.1-71.6)	71.5 (69.1-73.8)	53.7 (52.0-55.4)
Income (minimum wage per capita)							
< 2	2,077	16.9 (16.7-17.1)	15.5 (15.2-15.8)	72.1 (71.4-72.8)	68.9 (68.3-69.5)	69.7 (68.8-70.5)	52.7 (52.1-53.3)
2-3.9	926	16.9 (16.7-17.1)	16.7 (16.3-17.0)	76.3 (75.3-77.2)	68.9 (68.3-69.5)	69.7 (68.8-70.5)	61.2 (60.4-62.1)
4+	412	18.9 (18.5-19.3)	16.8 (16.2-17.4)	76.2 (74.8-77.6)	72.0 (70.7-73.3)	72.2 (70.5-73.9)	65.4 (64.1-66.7)

* Totals vary according to number of missing;

** Scores: 0-25;

*** Scores: 0-100;

^#^ Occupations that require special training and skills.

Table 2 shows that the following participants had substantially higher mean scores of satisfaction with their own health and quality of life in all its domains: those without mental distress; in the low-strain-at-work group; those who had not experienced work accidents; who did not report discrimination in the last 12 months at work, housing, public places, high school or college, or by the police; and who positively rated the relationship with their boss and co-workers.

**Table 2:** Means and 95% confidence intervals (95%CI) of the scores of satisfaction with one’s own health and quality of life (domains and extra-domain items), according to perception of discrimination, stressful events, work-related elements, and psychological distress. Pró-Saúde Study, 2001-2002.

	n *	Extra-domain ** [mean (95%CI)]			Domains *** [mean (95%CI)]		
		Overall quality of life	Satisfaction with health	Physical	Psychological	Social relationship	Environment
Job strain							
Low strain	1,200	18.2 (18.0-18.4)	16.7 (16.3-17.0)	76.4 (75.6-77.3)	72.3 (71.6-73.1)	72.7 (71.7-73.7)	59.5 (58.7-60.3)
Passive	907	17.5 (17.2-17.7)	15.9 (15.5-16.3)	73.3 (72.3-74.3)	68.8 (67.8-69.7)	69.7 (68.5-70.8)	59.5 (58.7-60.3)
Active	836	17.7 (17.4-18.0)	16.2 (15.8-16.6)	73.9 (72.9-74.9)	71.3 (70.4-72.2)	72.2 (70.9-73.4)	57.5 (56.5-58.5)
High strain	493	15.9 (15.4-16.3)	14.1 (13.5-14.7)	73.9 (72.9-74.9)	64.5 (63.1-65.9)	64.0 (62.1-65.8)	49.8 (48.5-51.1)
Social support at work							
Low	903	16.4 (16.1-16.8)	14.7 (14.3-15.1)	67.5 (66.4-68.6)	64.7 (63.6-65.7)	63.3 (61.9-64.6)	51.8 (50.8-52.8)
Fair	1,457	17.7 (17.5-17.9)	16.3 (16.1-16.6)	74.8 (74.0-75.5)	70.7 (70.0-71.4)	70.4 (69.5-71.3)	57.0 (56.3-57.7)
High	1,144	18.3 (18.1-18.6)	16.7 (16.3-17.0)	77.6 (76.8-78.5)	73.7 (72.9-74.5)	76.5 (75.5-77.6)	60.1 (59.3-60.9)
Accidents at work ^#^							
Yes	885	16.8 (16.5-17.1)	14.8 (14.4-15.2)	69.7 (68.7-70.8)	67.1 (66.1-68.2)	68.0 (66.7-69.2)	53.1 (52.1-54.0)
No	2,326	17.9 (17.7-18.1)	16.6 (16.4-16.8)	75.7 (75.1-76.3)	71.3 (70.8-71.9)	71.6 (70.8-72.3)	58.3 (57.8-58.9)
Stressful events ^##^							
1 or more	1,063	17.0 (16.7-17.3)	15.1 (14.7-15.5)	70.9 (69.9-71.9)	68.5 (67.6-69.5)	68.5 (67.4-69.7)	54.6 (53.7-55.5)
None	2,511	17.8 (17.6-18.0)	16.4 (16.1-16.6)	74.9 (74.3-75.5)	70.6 (70.1-71.2)	71.3 (70.6-72.0)	57.4 (56.8-58.0)
Discrimination ^#^							
Yes	563	16.1 (15.7-16.5)	14.3 (13.8-14.8)	66.9 (65.5-68.2)	65.4 (64.1-66.7)	63.1 (61.4-64.8)	49.3 (48.1-50.5)
No	2,976	17.8 (17.7-18.0)	16.3 (16.1-16.5)	75.0 (74.5-75.5)	70.9 (70.4-71.4)	71.9 (71.3-72.6)	58.0 (57.5-58.5)
Psychological distress ^###^							
Absent	2,369	18.5 (18.4-18.7)	17.2 (17.0-17.4)	78.5 (78.0-79.1)	75.0 (74.5-75.4)	75.0 (74.4-75.7)	59.6 (59.0-60.1)
Mild	769	15.9 (15.6-16.3)	14.0 (13.6-14.4)	65.7 (64.7-66.6)	62.2 (61.4-63.1)	62.0 (60.7-63.3)	51.7 (50.7-52.6)
Moderate/Severe	250	13.5 (12.8-14.3)	10.5 (9.8-11.3)	52.7 (50.8-54.6)	47.2 (45.3-49.1)	53.1 (50.4-55.8)	43.3 (41.5-45.1)

* Totals vary according to number of missing;

** Scores: 0-25;

*** Scores: 0-100;

^#^ Within 12 months prior to interview;

^##^ Hospital admission, robbery, physical assault, gunshot or weapon injury, traffic accident, within 12 months prior to interview;

^###^ Based on *General Health Questionnaire* (GHQ-12) scores from 0 to 36 (sum of Likert scale).

Table 3 shows that the mean scores of quality of life (overall and within domains) and satisfaction with health were higher among participants who reported leisure-time physical activity, fruit and vegetable consumption one or more times a week, and who did not report impediment to routine activities due to health problems in the two weeks prior to completing the questionnaire. Scores were also higher among those who did not have any of the selected chronic diseases, although the difference was rather small for overall quality of life and social relationship domain. The mean scores showed a slight downward trend according to smoking (never smoked, quit smoking, and current smokers) for satisfaction with health and quality of life domains, except in the social relationship domain, in which there was no difference. Regarding alcohol consumption, the differences in the mean scores of quality of life and satisfaction with health were negligible.

**Table 3:** Means and 95% confidence intervals (95%CI) of the scores of satisfaction with one’s own health and quality of life (domains and extra-domain items), according to lifestyle, behavior, and health status. Pró-Saúde Study, 2001-2002.

	n *	Extra-domain ** [mean (95%CI)]			Domains *** [mean (95%CI)]		
		Overall quality of life	Satisfaction with health	Physical	Psychological	Social relationship	Environment
Physical activity ^#^							
Yes	1,539	18.2 (18.0-18.4)	17.0 (16.7-17.3)	75.5 (74.8-76.3)	71.7 (71.0-72.4)	71.5 (70.6-72.4)	58.5 (57.8-59.3)
No	1,899	17.1 (16.8-17.3)	15.2 (15.0-15.5)	72.3 (71.6-73.0)	68.8 (68.1-69.4)	69.8 (68.9-70.7)	55.3 (54.6-55.9)
Eating habits							
Fruits and vegetables ≤ 3x month	269	16.4 (15.8-17.1)	15.1 (14.3-15.8)	71.0 (69.0-72.9)	65.0 (63.0-67.1)	65.0 (62.3-67.7)	53.5 (51.6-55.3)
Fruits or vegetables ≥ 1x week	666	17.2 (17.0-17.4)	15.8 (15.5-16.0)	73.1 (72.5-73.8)	69.4 (68.8-70.0)	69.6 (68.8-70.4)	55.4 (54.8-56.0)
Fruits and vegetables ≥ 1x week	2,606	18.3 (18.1-18.6)	16.5 (16.2-16.9)	75.1 (74.3-76.0)	72.1 (71.3-72.9)	73.1 (72.1-74.1)	59.2 (58.4-60.0)
Smoking							
Current smoker	752	16.9 (16.6-17.2)	15.2 (14.8-15.6)	72.1 (71.0-73.3)	69.2 (68.1-70.3)	70.4 (68.9-71.8)	55.4 (54.4-56.5)
Ex-smoker	778	17.5 (17.2-17.9)	15.6 (15.2-16.1)	72.7 (71.5-73.8)	69.7 (68.8-70.7)	70.2 (68.8-71.5)	56.4 (55.4-57.5)
Never smoked	1,924	17.8 (17.6-18.0)	16.4 (16.1-16.7)	74.7 (74.0-75.3)	70.4 (69.8-71.0)	70.6 (69.8-71.4)	57.1 (56.5-57.8)
Alcohol consumption (days) ^##^							
None	1,676	17.7 (17.5-17.9)	15.8 (15.5-16.1)	73.1 (72.3-73.9)	69.7 (69.0-70.4)	70.3 (69.4-71.2)	56.8 (56.1-57.5)
1-5	1,485	17.5 (17.3-17.7)	16.2 (15.9-16.5)	74.5 (73.8-75.3)	70.5 (69.8-71.2)	70.6 (69.6-71.5)	56.6 (55.9-57.4)
6-14	286	17.1 (16.5-17.7)	15.7 (15.0-16.4)	73.1 (71.3-75.0)	69.2 (67.4-70.9)	70.3 (68.0-72.7)	55.8 (54.0-57.6)
Recent morbidity ^###^							
None	2,468	18.1 (17.9-18.2)	17.0 (16.7-17.2)	77.3 (76.8-77.9)	72.0 (71.5-72.5)	72.4 (71.7-73.1)	58.6 (58.0-59.1)
≥ 1; did not seek care	650	15.3 (14.5-16.0)	13.4 (12.5-14.2)	64.5 (62.3-66.7)	62.4 (60.2-64.6)	62.8 (59.9-65.8)	49.4 (47.5-51.4)
≥ 1; sought care	183	16.2 (15.9-16.6)	12.7 (12.2-13.2)	61.9 (60.6-63.2)	64.3 (63.1-65.5)	65.5 (63.9-67.1)	51.6 (50.5-52.8)
Noncommunicable chronic diseases							
At least one	797	16.8 (16.5- 17.2)	13.6 (13.2-14.0)	68.6 (67.4-69.7)	67.1 (66.0-68.1)	68.8 (67.4-70.1)	54.3 (53.3-55.3)
None	2,392	17.8 (17.6-18.0)	16.7 (16.5-16.9)	75.3 (74.7-75.9)	70.8 (70.3-71.4)	70.8 (70.1-71.6)	57.5 (56.9-58.0)

* Totals vary according to number of missing;

** Scores: 0-25;

*** Scores: 0-100;

^#^ Within 12 months prior to interview;

^##^ Days of alcohol consumption within two weeks prior to interview;

^###^ Impediment of usual activities due to health problems within two weeks prior to interview.

### Structural equation model

In the final structural equation model, psychological distress, social support at work, demand at work, control at work, and discrimination showed significant association to all domains of quality of life, whereas socioeconomic status and noncommunicable chronic diseases did not remain in the social relationship model (Box 1). Stressful events and recent morbidity remained only in the final physical and environmental domains. Our final model showed a reasonable fit, meeting one of the goodness-of-fit indices (RMSEA) and performing closely to the cut-off points on the others: RMSEA = 0.54 (90%CI: 0.52-0.57), CFI = 0.890, TLI = 0.842, and SRMR = 0.091. The construct validity for the two latent variables of the model was confirmed based on factor loadings above 0.5 (Table 4).

**Box 1:** Exposure variables that remained in each equation of the final structural equation model.

QUALITY OF LIFE DOMAINS			
Physical	Psychological	Social relationship	Environmental
Psychological distress	Psychological distress	Psychological distress	Psychological distress
Noncommunicable chronic diseases	Noncommunicable chronic diseases		Noncommunicable chronic diseases
Socioeconomic status	Socioeconomic status		Socioeconomic status
Social support at work	Social support at work	Social support at work	Social support at work
Demand at work	Demand at work	Demand at work	Demand at work
Control at work	Control at work	Control at work	Control at work
Discrimination	Discrimination	Discrimination	Discrimination
Stressful events			Stressful events
Recent morbidity			Recent morbidity

**Table 4:** Standardized factor loadings, standard errors, and p-values of the latent variables that make up the model.

	Standardized factor loading (SE)	p-value
Socioeconomic status		
Per capita household income	0.699 (0.013)	< 0.001
Schooling	0.805 (0.015)	< 0.001
Job/Occupation	0.661 (0.014)	< 0.001
Discrimination		
At the workplace	0.503 (0.034)	< 0.001
On matters pertaining to housing	0.647 (0.045)	< 0.001
By the police	0.568 (0.039)	< 0.001
In public places	0.862 (0.033)	< 0.001
In high school or college	0.649 (0.039)	< 0.001

SE: standard error.

**Figure 2:**
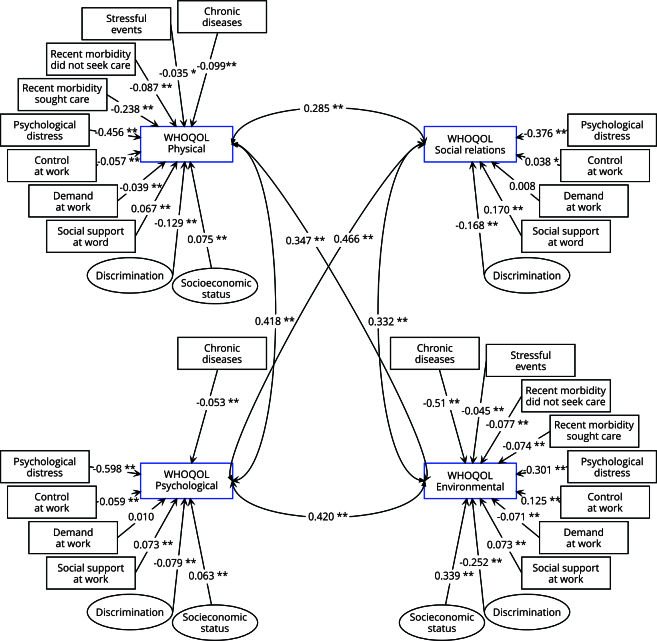
Standardized coefficients for exposure variables included in each of the four dimensions of quality of life in the final model.

Note: the level of significance is signaled by * p < 0.05, ** p < 0.01. Outcomes are outlined in blue. Confounding variables were gender, age, physical activity, and eating habits.

Figure 2 shows the standardized coefficients (SC) and their level of statistical significance for all exposures included in each equation of the final model. As there were four equations (one for each quality of life domain), the outcome variables (outlined in blue) are presented with their respective exposures, their standardized factor loadings, and statistical significance, alongside the correlations between each of the four quality of life domains. Although omitted, all equations included the four confounding variables already detailed (gender, age, physical activity, and eating habits).

The magnitude of association between psychological distress and all quality of life domains stood out from other covariates, particularly in the psychological domain (SC = -0.598, p < 0.01). The association between socioeconomic status and the environmental domain (SC = 0.339, p < 0.01) when compared to the other quality of life domains was also noteworthy. The negative association of recent morbidity leading to search for health care in the physical domain also had a relevant magnitude compared to other exposures (SC = -0.238, p < 0.01). Experiencing discrimination was negatively associated with the environmental domain (SC = -0.252, p < 0.01), whereas the magnitude of the association was lower for the other domains. A modest association was shown between social support at work and the social relations domain (SC = 0.170, p < 0.01), and between control at work and the environmental domain (SC = 0.125, p < 0.01).

## Discussion

The analyses showed variable degrees of association of several, observed and latent, variables with quality of life measured by the WHOQOL-BREF in civil servants at university campuses in Rio de Janeiro. The associations are aligned with the logical relationship of the variables with the concept of quality of life underlying the structured domains of the WHOQOL-BREF. Data also showed a correlation between the extra-domain scores - overall quality of life and satisfaction with health - and the domain scores, which has also been considered as indicative of construct validity ^21^. Our structural equation model contributed to the understanding of the construct quality of life by testing pathways previously theorized in qualitative studies and others not analyzed simultaneously in a single model. Furthermore, our model considered existing correlations among quality of life domains, improving measurement errors and providing robust estimates. Finally, our model showed a reasonable fit, specially considering its numerous variables and sample size, and it yielded plausible estimates of known associations.

The challenge is to distinguish variations that represent genuine differences in quality of life beyond the limitations of the scale. In this sense, higher income levels and education levels in individuals with higher scores in the quality of life domains have intuitive appeal. They are in line with a study on the general population in Pakistan ^22^, which found socioeconomic status as the major predictor of poor quality of life, and a metanalysis with a positive association of quality of life and human development index ^23^. Taken together, the factors that showed some degree of correlation may indicate the propensity to higher quality of life levels according to the characteristics of the individuals, such as lifestyle and socioeconomic status.

The results of this study suggest that quality of life scores might signal the need for further evaluation of individuals with very low scores for applications in clinical research and clinical practice ^24^. Since there are no established critical values to define “low scores” for the domains, the extra-domain items of the WHOQOL-BREF would be useful for screening individuals with an indication for in-depth evaluation. Therefore, WHOQOL-BREF could also be a tracer of vulnerability justifying further assessment for confirmation. Regarding these properties, quality of life measurement instruments can be applied to evaluate the direct and indirect results of interventions , as anticipated by the WHOQOL Group ^25^. With this purpose, one of the few reports in the literature was the evaluation of changes in quality of life following a leisure and physical activity program for older adults in two cities in the Brazilian state of Paraná, enabling the evaluation of a relevant aspect of public policies ^26^.

The association of the domains’ scores with other variables of the questionnaire can be interpreted by analyzing the facets that make up each domain, in which living conditions in general, aspects of the work environment, access to leisure, health care, and physical and mental well-being are recognized. In this last aspect, the overlapping of several facets of the WHOQOL-BREF domains with the GHQ-12 components is evident, explaining part of the outstanding inverse association with quality of life scores, and possibly indicating that the perception of quality of life was influenced by the emotional state. Heinonen et al. ^27^ warned of “contamination” by depressive symptoms in measures of quality of life. Chang et al. ^28^ also indicated that the association between quality of life and its determinants seemed to be affected by depressive symptoms in non-institutionalized older adults. However, Berlim et al. ^29^ concluded that the WHOQOL-BREF was reliable and valid in patients with major depression, and that treatment of depression improved quality of life scores. Considering the cross-sectional nature of the data, the association between mental distress and quality of life could be bidirectional, as both had the previous two weeks as a reference and the items that make up the quality of life domains can be determinants of mental distress.

Nevertheless, aspects such as discrimination, eating habits, and stressful events, which are not directly tackled in the facets of the WHOQOL, have implicit elements that can be indirectly related to quality of life. The association of socioeconomic status with the environmental domain, which includes directly related facets (e.g., financial resources), is worth highlighting. The variable recent morbidity showed a modest association, only with the physical domain, which has several of its facets aimed at the repercussions of recent morbidity on quality of life. This was not the case with the latent variable discrimination, of which the moderate association with the environmental domain did not seem to result from a direct relationship with some of the facets. The weak association of eating habits only with the psychological domains and social relationships (estimates not shown) is consistent with the non-directly related aspects captured in the facets of those domains. The association of demand, control, and social support at work was modest, but substantially higher with the social relationships domain than with others, consistent with the content of their facets. Part of the effect of work stress on quality of life may have been expressed as mental distress, considering the demonstrated association between exposure to stressful work conditions and mental health ^30^ ^,^ ^31^.

Some of the variables associated with quality of life pointed out by other authors, such as gender, age, and physical activity, did not show relevant independent associations among the Pró-Saúde Study participants. However, they had shown association with at least one of the domains in univariate analysis (Tables 1, 2, and 3), and only alcohol consumption was not associated to any of the quality of life domains. This may have occurred because of low variability of attributes, since the correlation may appear weak even if a conceptual relationship is already recognized in samples that are homogeneous or are approached with instruments unable to capture relevant differences between respondents in some variables. Some overlapping among attributes, such as chronic diseases and recent morbidity, stressful events and psychological distress, and job demands and discrimination may also have affected the final model. Moreover, for some variables such as recent morbidity, physical activity, and alcohol consumption, the recall period of two weeks may have missed differences among individuals, and thus, reduced variability. As validity should concern the inference based on the measurement, rather than the instrument itself, it may differ depending on the variable of interest for correlation assessment ^12^ ^,^ ^32^. Therefore, limited accuracy of covariates ascertained with instruments “calibrated” for purposes different from those of this study may have affected estimates of correlation. The suboptimal reproducibility of scores is also expected to attenuate estimates of correlation. However, test-retest analysis of measurements in Pró-Saúde Study ^7^ had showed that quality of life domains and GHQ-12 scores had high reliability.

The cross-sectional approach also hindered the analysis of the correlation between changes over time in quality of life and changes in the attributes included in the model (characterizing responsiveness as a dimension of sensitivity). Our analyses show caveats related to the imbedding of WHOQOL-BREF as the penultimate section of an 83-item questionnaire of the Pró-Saúde Study. It seems plausible that after having reflected on the multiple aspects that affect health and quality of life, the respondents had had opportunities to elaborate their answers. Another caveat concerns the assessment of mental distress by GHQ-12, which had also been applied in the first wave of the Pró-Saúde Study. Kalton & Citro ^33^ showed the potential for bias that the effect of participation in successive “waves” can cause in the responses to the items, as the participant gains familiarity with the questions. An analogous effect has been called the “practice effect” referring to the improvement in performance (or accuracy) on tests attributed to previous exposure to the same test ^34^. Moreover, interventions or referrals that may have occurred because of the identification of risk situations in wave 1 of Pró-Saúde Study (e.g., morbid obesity and very high GHQ-12 scores) might have artificially altered the scenario in the second wave.

A major strength of this study is its basis on a working population, comprising individuals of different job categories and who are healthy enough to work. This differs from studies in health services, which have quality of life as a secondary aspect. The measurement of quality of life in the Pró-Saúde Study, which investigated social determinants of health, enabled interpretations that are more connected with the comprehensive character of WHOQOL-BREF.

Instruments and scales are convenient to summarize, quantify, and explain theoretical constructs and structure the measurement of subjective attributes, aiming for greater homogeneity of understanding and interpretation of constructs, and maximum comparability of measures and classifications. Simplification attained by scales seems to be an acceptable trade-off for the subtleties in classifications and inferences from individualized measurements. The interpretation of scales implies, in a way, reversing the mental process that led to the content validation of the synthesis measure and spelling out the components that generated it.

Construct validation is a continuous process of testing hypotheses structured on the theory that underlies the construct and requires empirical verification of the performance of measurement instruments in different contexts ^12^. The construct validity of the WHOQOL-BREF based on factor analysis performed at the Pró-Saúde Study had shown that the factor structure did not replicate the results of the WHOQOL Group ^7^. The insufficient demarcation between the physical and the psychological domains and social relationships, “contaminated” by issues from other domains, was attributed, in part, to the characteristics of the Pró-Saúde Study participants. Nevertheless, the environmental domain showed performance like the WHOQOL-BREF field tests. Another study of Brazilian workers in a research institution also concluded that the psychological, general, and environmental domains were not compatible with the theoretical proposition of the WHOQOL Group and proposed the semantic revision and substitution of some items to improve the validity of the theoretical constructs ^35^. One might also ponder that changes in items of the scale should be considered but not be driven by factor analysis, so as to safeguard the comparability of results across studies. The aim of the scale is inferential, which depends more on content validity ^12^. The WHOQOL-BREF had already made concessions to content validity because of the reduction in the number of items from the WHOQOL-100, especially in the domains of independence and spirituality/religion/personal beliefs.

In conclusion, the results strengthened the evidence of construct validity from factor analysis of WHOQOL-BREF, with additional evidence inferred from association with other constructs, related to quality of life in different ways. The use of WHOQOL-BREF can provide valid inferences on the quality of life of workers, targeting very low scores that might signal the need for support, and assess changes in quality of life following interventions. A caveat worth considering comes with its utilization in parallel with other instruments for data collection tackling related aspects and, thus, influencing respondents’ perception of their quality of life.

## Data Availability

The research data are available upon request to the corresponding author.
