# Supplementary material for: Health, social, behavioral, and labor correlates of quality of life in a well population: a contribution to construct validity of the WHOQOL-BREF
Source: Cad Saude Publica. 2025 Dec 1;41(11):e00043325. doi: 10.1590/0102-311XEN043325 (PMC12688310; doi:10.1590/0102-311XEN043325)
Supplement: Supplementary Material [file 1678-4464-csp-41-11-EN043325-s.pdf]

SUPPLEMENTARY MATERIAL

Health, social, behavioral and labor correlates of quality of life in a well population: a contribution to construct validity of the WHOQOL-BREF

Submitted to Applied Research in Quality of Life

Supplementary Information (SI)

Methods to derive the covariates *noncommunicable chronic diseases*, *recent morbidity*, *job strain*, *participant's experience of stressful events in the previous 12 months*, *eating habits*, *discrimination* and *socioeconomic status* from specific questionnaire items.

Different from quality of life and its domains in WHOQOL-BREF, mental distress had a reference (full psychiatric evaluation) that allowed the evaluation of GHQ-12' criterion validity. In the standard classification, each item on the Likert scale (ordinal scale from 0 to 3) was dichotomized into 0 or 1 (absent or present) and the sum of 3 or more points in all items is classified as mental distress (Hair et al., 2019). Alternatively, the score was also be expressed on a scale of 0 to 36 (sum of the 12 items' scores on the Likert scale), with a score of 12 or more achieving better accuracy in detecting depression (Lundin et al., 2016). We also categorized the GHQ-12 Likert scale scores as "no suffering" (scores 0 to 12), "mild suffering" (13 to 20) and "moderate/severe suffering" (21 to 36).

A variable *Noncommunicable chronic diseases* was derived from the presence or history of hypertension, diabetes, myocardial infarction, emphysema/chronic bronchitis and stroke that had been informed to the patient by a health professional. The categories of chronic diseases were: at least one, none of them, and missing (no information on at least one of the diseases).

A variable *Recent morbidity* combined (1) health problems in the two weeks prior to filling out the questionnaire, which prevented them from carrying on usual activities (work, study, leisure, or household chores), and (2) seeking care for the main health problem mentioned. The categories of recent morbidity generated three dummy variables: (i) no morbidities, (ii) morbidities and did not seek care, and (iii) morbidities and sought care.

Job strain was assessed according to the demand-control model proposed by Robert Karasek (1979), whose instrument – the Demand, Control, Social Support Questionnaire (DCSQ) – was cross-culturally adapted for the Brazilian Portuguese (Alves et al., 2004; Alves et al., 2015). This

17-item scale encompasses three dimensions (demand, control, and social support), the first with five items and the others with six items. The items on the 4-point Likert scale range from "often" (1) to "never or almost never" (4). The score of each dimension is the sum of the scores of each item, ranging from 5 to 20 (demand) and from 6 to 24 (others). The higher the score, the lower was the construct grade. For each dimension of demand-control model, a dummy variable was created, with a value of 1 when the interviewee's score is lower than the median score of all participants, and zero if not, and, based on these, job strain was categorized according to the quadrant formulation proposed by Karasek (1979): low strain (low demand/high control); active (high demand/high control); passive (low demand/low control); and high strain (high demand/low control). Social support at work (respondent's perception of the relationship with the boss and co-workers) had the scores empirically categorized as "favorable" (6-17), "medium" (18-21) and "unfavorable" (22-24).

The participant's experience of stressful events in the previous 12 months was assessed by six questions about (i) hospitalization for one night or more, (ii) assault or theft of money or some property, (iii) physical assault, injury with a firearm or bladed weapon, suffered (iv) or witnessed (v) by the participant, and (vi) traffic accident (as a driver, passenger or pedestrian). Stressful events combining those 6 items were coded as 1 (at least 1 event), 0 (none of the events) and missing (missing data for 1 or more items).

A variable summarizing eating habits was derived from two items of the questionnaire on the consumption of vegetables, greens and fruits. The five categories of the two variables, ordering the frequency of consumption from "Never to at least once a month" to "Daily", were combined into one variable with three categories that captured the extremes and an intermediate level of consumption of those vegetables. Three *dummy* variables were created: (i) "worst" (vegetables and fruits less than 1 to 3 times/week), (ii) "medium" (vegetables **or** fruits 1 to 3 times or more/week), and (iii) "better" (vegetables **and** fruits 1 to 3 times or more/week.

The variables *discrimination* and *socioeconomic status* were analyzed as latent variables derived by factor analysis. Socioeconomic status was derived from the participant's schooling and occupation and the per capita income of the respondent's family. The perception of discrimination based on race/color, sex, age, religion, physical disease/disability, physical appearance, sexual orientation, economic condition, level of education or political activity, was based on reports in the last 12 months, combining the 5 scenarios addressed in the questionnaire: workplace, housing, public places, high school or college, or contact with the police. The construct validity for the latent variables *discrimination* and *socioeconomic status* was verified from factor loadings above 0.5.

The analyses also included the practice of any physical activity in the two previous weeks, to improve health, physical condition or for aesthetic or leisure purposes, and the occurrence of any

work related accidents in the previous 12 months (the questionnaire presented a list and whether they led to search for health care and to time away from work). Race and marital status were not ascertained in the second wave of Pró-Saúde Study.

References

1. Alves, M.G.M., Chor, D., Faerstein, E., Lopes, C.S., Werneck, G.L. (2004). Versão resumida da "Job Stress scale": adaptação para o português. Revista de Saúde Pública,38(2), 164-71. https://doi.org/10.1590/S0034-89102004000200003.

2. Alves, M.G.M., Braga, V.M., Faerstein, E., Lopes, C.S., Junger, W. (2015). Modelo demanda-controle de estresse no trabalho: considerações sobre diferentes formas de operacionalizar a variável de exposição. Cadernos de Saúde Pública. 31(1), 208-212. https://doi.org/10.1590/0102-311x00080714.

3. Hair, J.F., Black, W.C., Babin, B.J., Anderson, R.E., Tatham, R.L. (2019). Multivariate Data Analysis. 8th ed. Cengage Learning EMEA.

4. Karasek, R.A. (1979). Job demands, job decision latitude, and mental strain: Implications for job redesign. Administrative Science Quarterly, 24(2), 285–308. https://doi.org/10.2307/2392498.

5. Lundin, A., Hallgren, M., Theobald, H., Hellgren, C., Torgén, M. (2016). Validity of the 12-item version of the General Health Questionnaire in detecting depression in the general population. Public Health, 136, 66-74. https://doi.org/10.1016/j.puhe.2016.03.005.
